# A Giant Lipoma Presenting With Neurological Symptoms: A Rare Clinical Presentation

**DOI:** 10.7759/cureus.84734

**Published:** 2025-05-24

**Authors:** Partha Nandi, Dhananjay Ahlawat

**Affiliations:** 1 Department of General Surgery, Noida International Institute of Medical Sciences, Greater Noida, IND

**Keywords:** excision, giant lipoma, intermuscular lipoma, lipoma, surgery

## Abstract

A lipoma is the most common benign soft tissue tumour. Also known as ‘universal tumours’, lipomas can occur anywhere where there is adipose tissue. Most of them do not require surgical intervention. However, an increase in size, pain and cosmetic deformity might require surgical excision. A sudden increase in size might be secondary to malignant transformation, and hence any suspicion requires histopathological evaluation. This report presents the case of a 54-year-old female patient who was diagnosed with a giant intramuscular lipoma, causing neurological symptoms. She underwent excision for the same under spinal anaesthesia.

## Introduction

A lipoma constitutes the most common soft tissue tumour [1]. They can either be subcutaneous and subfascial or intermuscular and intramuscular. Intramuscular lipomas originate from between the muscle fibres and penetrate adjacent muscle, passing through the intermuscular septa [2, 3]. Giant lipomas measure at least 10 cm in diameter or should have a minimum weight of 1000  g [4]. Giant lipomas account for only 1% of all lipomas [5]. Lipomas are asymptomatic, but based on their location and size, they can cause nerve compression leading to neurological symptoms [6]. Here, we present a case of a 54-year-old female patient who presented with radiating low backache and later on got diagnosed with intramuscular lipoma with neurological symptoms.

## Case presentation

A 54-year-old woman presented to the surgical outpatient department with a chief complaint of persistent radiating low backache for the last year, which used to get relieved with over-the-counter oral analgesics. She further complained that she felt pain over her left lower back while lying down, with radiation to her left leg, with a tingling sensation.

On examination, a vague, ill-defined lump was felt over her left lower back, approximately 5 cm x 5 cm in size, partially mobile, non-tender and deep-seated. However, she complained of discomfort over that area when pressed during examination. Neurologically, no motor deficit was noted, but she persistently complained of radiating pain and on-and-off tingling over the said limb. An ultrasound of the soft tissue was done, which was suggestive of an intramuscular lipoma located near the L5-S1 vertebra on the left side. An X-ray of the lumbosacral (LS) spine was done, and the orthopedician's opinion was taken, which was overall normal. An MRI of teh LS spine was prescribed, but the patient opted out because of affordability. Fine needle aspiration cytology (FNAC) was done to confirm the cytology of the soft tissue tumour, and it was found to be a lipoma.

**Figure 1:**
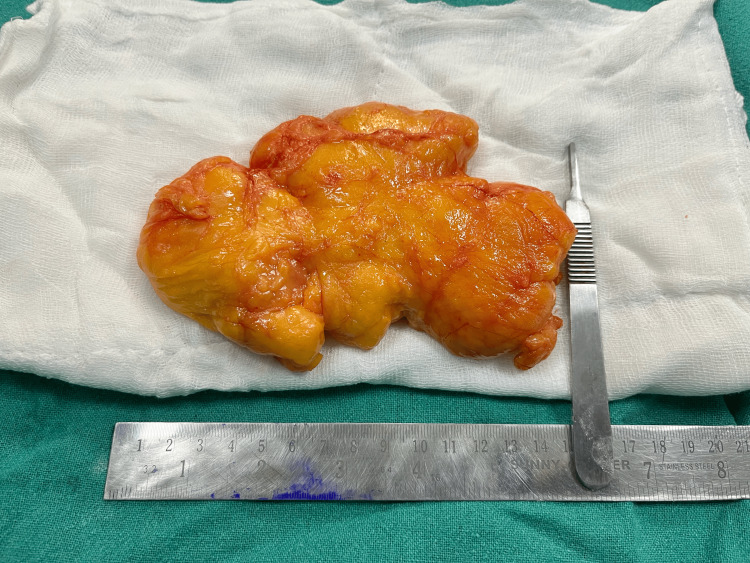
Intramuscular giant lipoma (15 cm in its maximum dimension)

**Figure 2:**
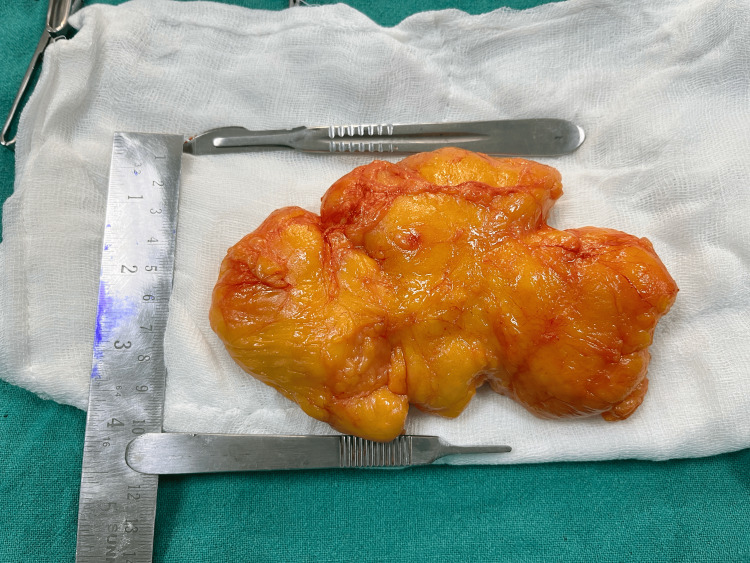
Intramuscular lipoma (10 cm in width)

The patient was admitted for surgery. Her vitals were stable with a pulse rate (PR): 82 beats per minute (bpm), blood pressure (BP): 110/68 mmHg, oxygen saturation (SpO₂): 97% in room air. Haematological parameters, ECG, and chest X-ray were within normal limits. After a preanaesthetic checkup, she was taken up for surgery under spinal anaesthesia. A 15 cm x 10 cm lipoma with intramuscular extensions was excised (Figure 1 and Figure 2). Postoperatively, she recovered well and was discharged on the second postoperative day. On follow-up, there was complete resolution of symptoms.

## Discussion

Lipomas remain the most common mesenchymal tumour of the human body. About one in every 1000 people ends up having lipomas in their lifetime. The most common locations are the trunk and upper extremity, but they can appear anywhere. The cause of lipoma, however, remains unknown [7]. Histology reveals mature, normal-appearing adipocytes with a small eccentric nucleus intermixed among thin fibrous septa containing blood vessels [8].

Clinically, lipomas are soft, solitary, painless, mobile lumps often subcutaneous in location. The characteristic "slip sign" may be elicited. They are typically slow-growing and may grow over the years. A rapid increase in size must evoke suspicion towards malignant transformation.

Lipomas are mostly seen as cosmetic or physical deformities, but at times, due to their size and weight, they can show signs of compression. Neurological manifestations can rarely be brought on by a lipoma sitting next to a nerve [9].

In this case, owing to the presenting symptoms, which were mostly compressive and neurological, an MRI of the LS spine was prescribed. But the patient could not afford the investigation, and hence an X-ray of the LS spine along with USG soft tissue was done. FNAC was prescribed after the USG report, which was suggestive of a giant intramuscular lipoma. However, the treatment remains excision with the surrounding fibrous capsule to prevent recurrence.

## Conclusions

Lipoma, a common soft tissue tumour, mostly remains symptomless and requires no intervention. However, in certain conditions, a lipoma warrants detailed investigation. In case of a rapid increase in size or if it is a giant lipoma, further investigation, especially histopathological examination, is of utmost necessity. Mostly, it is the fear of malignancy or cosmetic deformity that brings the patient to a surgical OPD. But there are certain specific conditions where a lipoma presents with unusual symptoms like a radiating neurological pain. That happens mostly because of the location or the size of the lipoma, putting pressure on the adjacent structures. Especially, a lipoma over the lower back and in the paraspinal area might produce neurological symptoms like radiating pain. Hence, careful evaluation of such patients becomes necessary to rule out spinal or neurological causes. Excision remains the standard treatment for lipomas, and careful removal of the fibrous capsule is necessary to avoid recurrence.
